# Confidence Intervals for a Proportion Estimated From Pooled Samples Based on Firth's Corrected Score

**DOI:** 10.1002/bimj.70185

**Published:** 2026-09-28

**Authors:** Brad J. Biggerstaff, Graham Hepworth

**Affiliations:** ^1^ Division of Vector‐Borne Diseases Centers for Disease Control and Prevention Fort Collins, Colorado USA; ^2^ School of Mathematics and Statistics The University of Melbourne Melbourne, Victoria Australia

**Keywords:** binomial proportion, expected information, group testing, observed information, penalized likelihood, pooled testing, skewness correction

## Abstract

Interval estimation of a proportion estimated from testing of pooled samples has been studied by a variety of researchers, who have examined both exact and asymptotic methods. Recent work in point estimation has seen the use of Firth's correction to maximum likelihood estimation to reduce bias effectively. We consider the Firth‐corrected score statistic, using either expected or observed information, as the first derivative function of a penalized likelihood and develop score‐based confidence intervals wholly within this framework. We evaluate their performance, comparing them by information type and to the existing, recommended asymptotic method of Gart. The methods are illustrated using data on West Nile virus infection in field‐collected mosquitoes and on yellow fever virus infection in mosquitoes reported in Walter et al. The penalized likelihood approach using expected information is found to unify previously recommended point and interval estimates under one inferential framework. Detailed evaluation of coverage properties of the intervals over a range of pooling designs show improved performance for the intervals using observed information and skewness‐correction to the score. We derive simple, interpretable expressions for the score and skewness‐corrected score statistics, and give a quadratic formula for the skewness‐corrected score interval for the case that all pools are the same size, which reduces to a skewness‐correction for the standard Wilson interval in the standard binomial case.

## 1 Introduction

Pooled testing (or group testing) occurs when individual samples are pooled together and tested as a group for the presence of an attribute, usually a marker of infection. A positive test result indicates that at least one individual in the group is positive. Pooled testing for estimation of proportions has been applied in many areas, including transmission of viruses by insect vectors (e.g., Crockett et al. 2012), plant disease assessment (e.g., Liu et al. 2011), and HIV prevalence estimation (e.g., Zenios and Wein 1998). The obvious advantage of pooled testing, especially when the proportion is small, is the potential for considerable saving of resources, because fewer tests are needed in comparison to testing each sample separately.

The maximum likelihood estimator (MLE) of the population proportion p is positively biased. Burrows (1987) developed an estimator for equal pool sizes which removed nearly all the bias, and also reduced mean squared error. Hepworth and Watson (2009) successfully applied a general method of bias correction for the MLE described by Gart (1991) to the case of unequal pool sizes.

Firth (1993) introduced a general method of adjusting the MLE by modifying the usual score equation. The modification was motivated as a correction for bias in the estimating equation, implemented by subtracting the information‐scaled bias of the MLE from the score function. Here one may use either the expected (Fisher) information or the observed information, the latter for which Firth (1993) provided theoretical arguments for better, higher‐order performance. The scaled bias adjustment effectively re‐locates the estimating function with the maximum nearer the true parameter value, without the bias in the uncorrected score function. Throughout the paper, when we refer to “Firth's correction” we mean using either the expected or observed information, and we will otherwise specify which of these when needed.

For the problem of estimating p from pooled data with unequal sized pools and assuming perfect testing, Hepworth and Biggerstaff (2017) applied Firth's method with expected information to derive an estimator. They showed that this estimator is nearly unbiased for a range of pooling strategies or “designs” and of values of p encountered in practice. They also showed that Firth's method is equivalent to Burrows's method when applied to equal pool sizes.

For pooled testing, confidence interval (CI) estimation for p was first considered by Chiang and Reeves (1962) for equal‐sized pools or pools of two sizes, while the general case for unequal‐sized pools was first considered in Walter et al. (1980), who derived the MLE and its asymptotic variance, which they used for the standard Wald CI. In a series of papers, Hepworth (1996, 2004, 2005) investigated exact and asymptotic CIs for p in pooled testing for unequal‐sized pools. Among the asymptotic methods developed and evaluated in Hepworth (2005), a skewness‐corrected likelihood score interval derived using methods originally developed in Bartlett (1953) and subsequently employed by Gart (1991) was recommended based on coverage and expected length properties.

Noting as above that the Firth score adjustment effectively aligns and reshapes the estimating function to be maximized nearer the “true” value for p, we sought to develop improved CIs based on this adjustment, considering both expected and observed information. Firth (1993) discussed the interpretation of this introduced bias term in the context of penalized likelihood, for example, showing that it resulted in Jeffreys penalty in the case of an exponential family likelihood with the canonical link. Our approach follows this idea by treating the Firth‐corrected score functions using expected or observed information as arising from adoption of the corresponding penalized likelihood for inference, placing both point and interval estimation in a consistent inferential framework. Within these penalized likelihood frameworks, while we can develop the three usual likelihood‐based CIs—Wald, likelihood ratio test‐based, and score—we focus development and evaluation on those related to the score. Consistent with literature already cited and based on our own preliminary investigations, the general performance of the Wald‐type and likelihood ratio test based‐type CIs did not perform as well. When developing the score‐based intervals, we employ the skewness adjustments described in Gart (1991) to the developed penalized score‐based intervals, following justification given below. We compare the performances of the penalized score and skewness‐corrected score intervals using expected and observed information over a variety of pooled sampling designs. Throughout, only two‐sided CIs are considered.

When comparing CI performance by information type, one anticipates comparable performance based on an “overall” comparison, as (exact) coverage averages interval coverage over all possible outcomes weighted by their probability of occurrence. Since the average of the observed information is, by definition, the expected information, this averaging means that overall performance of observed and expected information‐based CIs should be similar. Of interest for full understanding of performance characteristics, then, is performance over outcomes which may not have relatively high probability of occurrence, but which nevertheless are possible. In these cases, observed information‐based CIs may be anticipated theoretically to perform better, since the observed information retains the observation in its computation, and so may better quantify the curvature of the penalized likelihood and corrected score function than the expected information for relatively improbable values. We therefore motivate and introduce the standardized information statistic as a tool to objectively partition the outcome space into sets over which we anticipate different performance characteristics for the expected and observed information‐based CIs. Finally, we summarize performance of the CIs over these sets to inform final recommendations on CIs for general use.

In Section 2 we introduce the model and existing, standard likelihood CI methods, including the skewness‐corrected CI of Gart (1991). Firth's correction and development of the penalized likelihood and associated CIs are developed in Section 3. In Section 4, we introduce two example applications, one drawn from an important pooled testing paper of Walter et al. (1980) that we use to illustrate the methods, and the other using data from surveillance for infection of mosquitoes with West Nile virus (WNV) in Fort Collins, Colorado, USA. Purpose‐written code for R statistical software (R Core Team 2023) was used for all computations and graphics. A detailed evaluation based on exact coverage properties of the CIs presented over a range of sample sizes and pooling configurations is given in Section 5, with concluding recommendations made in Section 5.4. We close the paper with a discussion in Section 6.

## 2 Model and Likelihood Methods

In this section we set the notation, review the model and existing likelihood‐based methods, and develop the penalized likelihood inferential framework and associated CIs.

Assume that there is a population of individuals either “positive” or “negative” for some characteristic of interest, and a random sample of size N individuals is selected. Letting p be the probability an individual is positive, to estimate p we first aggregate sampled individuals into groups or pools of d distinct sizes $m_{i}\in \left\{1,2,\cdots ,N\right\}$, for i=1,2,⋯,d. Suppose that there are $n_{i}$ pools of size $m_{i}$, so that $N=\sum_{i=1}^{d}m_{i}n_{i}=\sum_{i=1}^{d}N_{i}$. Rather than evaluating individuals, we determine positivity $Y_{ij}$ for each pool of size $m_{i}$, for each $j=1,2,\cdots ,n_{i}$ and i=1,2,⋯,d, using a perfect test or method, and set $Y_{ij}=1$ for a positive pool and $Y_{ij}=0$ for a negative pool. The distribution of the number of positive pools of size $m_{i}$, $X_{i}=\sum_{j=1}^{n_{i}}Y_{ij}$, is thus binomial $\left(n_{i},1-{\left(1-p\right)}^{m_{i}}\right)$, and a log‐likelihood for sample $x=(x_{1},x_{2},\cdots ,x_{d})$ is

$$\begin{array}{ccc} l(p;x)=l(p) & = & \sum_{i=1}^{d}x_{i}\log\left[1-{\left(1-p\right)}^{m_{i}}\right]\\ & & +\log\left(1-p\right)\sum_{i=1}^{d}m_{i}\left(n_{i}-x_{i}\right). \end{array}$$

Maximum likelihood estimation proceeds as usual by solving the score equation 

(1) $$\begin{array}{ccc} \frac{dl(p)}{dp} & = & S\left(p\right)=\frac{1}{1-p}\sum_{i=1}^{d}\left[\frac{m_{i}x_{i}}{1-{\left(1-p\right)}^{m_{i}}}-m_{i}n_{i}\right]\\ & = & \frac{1}{1-p}\sum_{i=1}^{d}\left[\frac{m_{i}x_{i}}{1-Q_{i}}-m_{i}n_{i}\right]=0, \end{array}$$

introducing for brevity the notation $Q_{i}={\left(1-p\right)}^{m_{i}}$; the solution $\hat{p}$ to this equation is thus the MLE. Using the Newton–Raphson algorithm, Walter et al. (1980) gave a simple, iterative expression to compute the MLE, and they derived its asymptotic variance $\text{Var}\left[\hat{p}\right]$ for $\hat{p}$ as the inverse of the Fisher information

$$\begin{array}{c} I\left(p\right)=\frac{1}{{\left(1-p\right)}^{2}}\sum_{i=1}^{d}\frac{m_{i}^{2}n_{i}Q_{i}}{1-Q_{i}}, \end{array}$$

computed from the observed information

(2) $$\begin{array}{ccc} i(p) & = & \frac{1}{{\left(1-p\right)}^{2}}\sum_{i=1}^{d}N_{i}\left\{\frac{x_{i}}{n_{i}\left(1-Q_{i}\right)}\left[\frac{\left(m_{i}+1\right)Q_{i}-1}{1-Q_{i}}\right]+1\right\}.\; \end{array}$$

(For readers familiar with Firth (1993), note that we reverse the capitalization of “i” for these quantities to be consistent with our previous work.) For implementation of methods due to Gart (1991) and Bartlett (1953), Hepworth (2005) derived the bias b(p) of $\hat{p}$, and the skewness $\mu_{3}\left(p\right)$ of the distribution of S(p), as

$$\begin{array}{c} b\left(p\right)=\frac{1}{2{\left(1-p\right)}^{3}{\left[I(p)\right]}^{2}}\sum_{i=1}^{d}\frac{m_{i}^{2}\left(m_{i}-1\right)n_{i}Q_{i}}{1-Q_{i}} \end{array}$$

and

$$\begin{array}{c} \mu_{3}\left(p\right)=\frac{1}{{\left(1-p\right)}^{3}}\sum_{i=1}^{d}\frac{m_{i}^{3}n_{i}Q_{i}\left(2Q_{i}-1\right)}{{\left(1-Q_{i}\right)}^{2}}. \end{array}$$

The score CI is given as the solutions for p in the equations

$$\begin{array}{c} Z\left(p\right)=\frac{S(p)}{\sqrt{I(p)}}=\pm z_{\alpha /2}, \end{array}$$

or, equivalently,

(3) $$\begin{array}{c} Z^{2}\left(p\right)=\frac{{\left(\sum_{i=1}^{d}N_{i}\left[\frac{x_{i}-n_{i}\left(1-Q_{i}\right)}{n_{i}\left(1-Q_{i}\right)}\right]\right)}^{2}}{\sum_{i=1}^{d}\frac{m_{i}^{2}n_{i}Q_{i}}{1-Q_{i}}}=z_{\alpha /2}^{2}, \end{array}$$

where $z_{\theta }$ is the 100(1−θ)th quantile of the standard normal distribution. Note that the score statistic Z(p) is asymptotically (in the $\left\{n_{i}\right\}$) standard normal, from the asymptotic normality of the binomial random variables $\left\{x_{i}\right\}$. When d=1 so that there is just one pool size, $m_{1}$, as shown just below these equations reduce to the standard score interval, (L,U), of Wilson (1927) for success probability $Q_{1}={\left(1-p\right)}^{m_{1}}$, from which the interval for p is computed directly as $\left(1-{\left(1-L\right)}^{1/m},1-{\left(1-U\right)}^{1/m_{1}}\right)$. If, further, $m_{1}=1$, then these intervals reduce to the standard binomial score interval for p (Wilson 1927). Setting $B=\left(z_{\alpha /2}^{2}-1\right)/6$, skewness‐corrected score intervals are found by solving the equations $Z\left(p\right)-B\;\gamma \left(p\right)=\pm z_{\alpha /2}$ or, equivalently,

(4) $$\begin{array}{c} \frac{{\left(\sum_{i=1}^{d}N_{i}\left[\frac{x_{i}-n_{i}\left(1-Q_{i}\right)}{n_{i}\left(1-Q_{i}\right)}\right]-B\;\;\frac{\sum_{i=1}^{d}\frac{m_{i}^{3}n_{i}Q_{i}\left(2Q_{i}-1\right)}{{\left(1-Q_{i}\right)}^{2}}}{\sum_{i=1}^{d}\frac{m_{i}^{2}n_{i}Q_{i}}{1-Q_{i}}}\right)}^{2}}{\sum_{i=1}^{d}\frac{m_{i}^{2}n_{i}Q_{i}}{1-Q_{i}}}=z_{\alpha /2}^{2}, \end{array}$$

where $\gamma \left(p\right)=\mu_{3}\left(p\right)/I{\left(p\right)}^{3/2}$. As with the uncorrected score case, when d=1, Equation (4) reduces to a quadratic equation in $Q_{1}$, and with ${\bar{p}}_{1}=x_{1}/n_{1}$, solutions ${\bar{Q}}_{1}$ are given by

(5) $$\begin{array}{ccc} {\bar{Q}}_{1} & = & \left[\left(1-\frac{2B}{n_{1}}\right)\left(1-{\bar{p}}_{1}-\frac{B}{n_{1}}\right)+\frac{z_{\alpha /2}^{2}}{2n_{1}}\right.\\ & & \;\pm \left.\frac{z_{\alpha /2}}{n_{1}}\sqrt{n_{1}\left({\bar{p}}_{1}-\frac{B}{n_{1}}\right)\left(1-{\bar{p}}_{1}-\frac{B}{n_{1}}\right)+\frac{z_{\alpha /2}^{2}}{4}}\;\;\right]\\ & & \;/\left[{\left(1-\frac{2B}{n_{1}}\right)}^{2}+\frac{z_{\alpha /2}^{2}}{n_{1}}\right]. \end{array}$$

This formula agrees, after simplification, with the standard skewness‐corrected score interval formula reported using the quadratic coefficients in the appendix of Laud (2017), though here for the more general, pooled case. Setting B=0 in these expressions gives the solutions to Equation (3) in this case, d=1, recognizable as the formula of Wilson (1927) for the confidence limits for the probability $Q_{1}$ that a pool of size $m_{1}$ is negative. CI limits for p are then found from $Q_{1}={\left(1-p\right)}^{m_{1}}$ as above for the uncorrected score. Likelihood‐based, asymptotic CIs were investigated in Hepworth (2005) and Biggerstaff (2008), and the generally recommended interval is the skewness‐corrected score method in Equation (4).

## 3 Firth's Correction and Penalized Likelihood

### 3.1 Expected Information

As MLEs are often biased, Firth (1993) introduced a general approach to bias reduction by adjusting the score estimating equation. Rather than solving Equation (1), he derived a modified estimation function to remove the first‐order bias by adjusting the score function by the bias of the MLE scaled by the local curvature (i.e., the information). As noted and discussed in Firth (1993), either expected or observed information may be used. Our development begins with Firth's modified score equation using the expected information. Set

(6) $$\begin{array}{c} \overset{˘}{S}\left(p\right)\equiv S\left(p\right)-I\left(p\right)b\left(p\right)=0, \end{array}$$

for which we denote the resulting solution $\overset{˘}{p}$. Hepworth and Biggerstaff (2017) developed this estimator under the pooled binomial model, gave a simple, iterative formula using the Newton–Raphson method for its computation, and investigated its performance.

To obtain a Firth penalized log‐likelihood function $\overset{˘}{l}\left(p\right)$ from the score $\overset{˘}{S}\left(p\right)$, compute

(7) $$\begin{array}{c} \overset{˘}{l}\left(p\right)=\int_{0}^{p}\overset{˘}{S}\left(u\right)\;du=l\left(p\right)-\int_{0}^{p}I\left(u\right)b\left(u\right)\;du, \end{array}$$

by the Fundamental Theorem of Calculus.

When all pools are the same size $m_{1}\equiv m$, say, this integral is available analytically:

$$\begin{array}{ccc} \overset{˘}{l}\left(p\right) & = & \int_{0}^{p}\overset{˘}{S}\left(u\right)du\\ & = & \int_{0}^{p}\left[S(u)-I(u)b(u)\right]du\\ & = & l\left(p\right)-\int_{0}^{p}\frac{1}{2}\frac{m-1}{1-u}du\\ & = & l\left(p\right)+\frac{1}{2}\left(m-1\right)\log\left(1-p\right)\\ & = & x\log\left[1-{\left(1-p\right)}^{m}\right]+\left[m\left(n-x\right)+\frac{1}{2}\left(m-1\right)\right]\log\left(1-p\right). \end{array}$$

Analytic computation of the penalized log‐likelihood $\overset{˘}{l}\left(p\right)$ for the more general case with different pool sizes $\left\{m_{i}\right\}$ is hampered by evaluation of the integral of the penalty term. Direct simplification results in the following expression:

$$\begin{array}{ccc} \overset{˘}{l}\left(p\right) & = & \int_{0}^{p}\overset{˘}{S}\left(u\right)du\\ & = & \int_{0}^{p}\left[S(u)-I(u)b(u)\right]du\\ & = & l\left(p\right)+\frac{1}{2}\log\left(1-p\right)-\frac{1}{2}\int_{0}^{p}\frac{1}{1-u}\sum_{i=1}^{d}m_{i}\;\left[\frac{m_{i}^{2}n_{i}U_{i}}{\sum_{j=1}^{d}m_{j}^{2}n_{j}U_{j}}\right]du \end{array}$$

with $U_{k}={\left(1-u\right)}^{m_{k}}/\left[1-{\left(1-u\right)}^{m_{k}}\right]$ for k=1,2,⋯,d. For evaluation of this quantity in application, one may use numerical integration in evaluations when direct computation of the penalized likelihood is required.

From its definition, we have that $E\left[\overset{˘}{S}\left(p\right)\right]=-I\left(p\right)b\left(p\right)$. Further, the central moments of $\overset{˘}{S}\left(p\right)$ are the same as those for S(p), since for k=1,2,⋯, $E\left[{\left(\overset{˘}{S}\left(p\right)-E\left[\overset{˘}{S}\left(p\right)\right]\right)}^{k}\right]=E\left[S{\left(p\right)}^{k}\right]$, as $E\left[S(p)\right]=0$. An expected information Firth penalized score statistic can thus be defined as

$$\begin{array}{c} \overset{˘}{Z}\left(p\right)=\frac{\overset{˘}{S}\left(p\right)-E\left[\overset{˘}{S}\left(p\right)\right]}{\sqrt{I(p)}}=\frac{S(p)}{\sqrt{I(p)}}, \end{array}$$

which is seen to match, computationally, the score statistic under the standard likelihood in Equation (3). We thus construct 100(1−α) percent penalized likelihood score‐based CIs by using the score‐based CI in Equation (3) and the skewness‐corrected score‐based CI in Equation (4). While these CIs agree computationally with the standard likelihood, they are derived adopting the expected information Firth penalized likelihood for inference.

### 3.2 Observed Information

As an alternative to using the expected information, begin with the Firth‐corrected score function using the observed information, and write $\overset{´}{S}\left(p\right)$ for this score function. Solving the estimating equation 

(8) $$\begin{array}{c} \overset{´}{S}\left(p\right)=S\left(p\right)-i\left(p\right)b\left(p\right)=0, \end{array}$$

with solution $\overset{´}{p}$ provides a point estimator for p (Firth 1993). Similar to above, the observed information Firth penalized log‐likelihood $\overset{´}{l}\left(p\right)$ may be obtained directly as

(9) $$\begin{array}{c} \overset{´}{l}\left(p\right)=\int_{0}^{p}\overset{´}{S}\left(u\right)\;du=l\left(p\right)-\int_{0}^{p}i\left(u\right)b\left(u\right)\;du, \end{array}$$

which requires numerical integration for evaluation.

We proceed to computing CIs by standardizing the score function $\overset{´}{S}\left(p\right)$, requiring the calculation of its mean and variance, and we further compute its skewness for correction in CI computation. With E[i(p)]=I(p), the mean is $\overset{´}{\mu }\left(p\right)=E\left[\overset{´}{S}\left(p\right)\right]=-I\left(p\right)b\left(p\right)$. For computation of the variance ${\overset{´}{\sigma }}^{2}\left(p\right)=\text{Var}\left[\overset{´}{S}\left(p\right)\right]$ and the skewness $\mu_{3}\left[\overset{´}{S}\left(p\right)\right]=E\left[{\left\{\overset{´}{S}\left(p\right)-E\left[\overset{´}{S}\left(p\right)\right]\right\}}^{3}\right]$, write

$$\begin{array}{cc} \overset{´}{S}\left(p\right)=\; & \sum_{i=1}^{d}\left\{\frac{m_{i}}{\left(1-p\right)\left(1-Q_{i}\right)}\left(1-\frac{m_{i}b\left(p\right)}{{\left(1-p\right)}^{2}}\left[\frac{\left(m_{i}+1\right)Q_{i}-1}{1-Q_{i}}\right]\right)x_{i}\right.\\ & \;\;\left.-\frac{m_{i}n_{i}}{1-p}\left[1+\frac{b(p)}{1-p}\right]\right\}, \end{array}$$

gathering the terms in $x_{i}$. Recalling that the variance and skewness of the sum of independent random variables are the sums of their individual respective values, and that $\text{Var}\left[x_{i}\right]=n_{i}Q_{i}\left(1-Q_{i}\right)$ and $\mu_{3}\left[x_{i}\right]=n_{i}Q_{i}\left(1-Q_{i}\right)\left(2Q_{i}-1\right)$, we directly compute

$$\begin{array}{cc} {\overset{´}{\sigma }}^{2}\left(p\right) & =\frac{1}{{\left(1-p\right)}^{2}}\sum_{i=1}^{d}\frac{m_{i}^{2}n_{i}Q_{i}}{1-Q_{i}}{\left[1-\frac{b(p)}{1-p}\frac{\left(m_{i}+1\right)Q_{i}-1}{1-Q_{i}}\right]}^{2} \end{array}$$

and

$$\begin{array}{cc} {\overset{´}{\mu }}_{3}\left(p\right) & =\frac{1}{{\left(1-p\right)}^{3}}\sum_{i=1}^{d}\frac{m_{i}^{3}n_{i}Q_{i}\left(2Q_{i}-1\right)}{{\left(1-Q_{i}\right)}^{2}}{\left[1-\frac{b(p)}{1-p}\frac{\left(m_{i}+1\right)Q_{i}-1}{1-Q_{i}}\right]}^{3}. \end{array}$$

Next define the observed information Firth penalized score statistic as the standardized version of $\overset{´}{S}\left(p\right)$,

$$\begin{array}{c} \overset{´}{Z}\left(p\right)=\frac{\overset{´}{S}\left(p\right)-\overset{´}{\mu }\left(p\right)}{\sqrt{{\overset{´}{\sigma }}^{2}\left(p\right)}}=\frac{S\left(p\right)-\left[i(p)-I(p)\right]b\left(p\right)}{\overset{´}{\sigma }\left(p\right)}. \end{array}$$

Asymptotic normality of $\overset{´}{S}\left(p\right)$ and i(p), and so of $\overset{´}{Z}\left(p\right)$ follows from the independence and asymptotic normality of the underlying binomial random variables, $\left\{x_{i}\right\}$. We then compute 100(1−α) percent observed information Firth penalized score CIs by solving the equations $\overset{´}{Z}\left(p\right)=\pm z_{\alpha /2}$, or, setting

$$\begin{array}{c} W_{i}\left(p\right)=1-\frac{b(p)}{1-p}\frac{\left(m_{i}+1\right)Q_{i}-1}{1-Q_{i}}, \end{array}$$

by solving

(10) $$\begin{array}{c} {\overset{´}{Z}}^{2}\left(p\right)=\frac{{\left(\sum_{i=1}^{d}N_{i}\left[\frac{x_{i}-n_{i}\left(1-Q_{i}\right)}{n_{i}\left(1-Q_{i}\right)}\right]W_{i}\left(p\right)\right)}^{2}}{\sum_{i=1}^{d}\frac{m_{i}^{2}n_{i}Q_{i}}{1-Q_{i}}W_{i}^{2}\left(p\right)}=z_{\alpha /2}^{2} \end{array}$$

for p. One observes here that when d=1 and so there is a single pool size, $m_{1}$, the expression for ${\overset{´}{Z}}^{2}\left(p\right)$ reduces to that for $Z^{2}\left(p\right)$ in Equation (3), as the $W_{1}\left(p\right)$ terms cancel. Thus, when there is just a single pool size, expected and observed information score intervals are exactly the same; further interpretation of this result will be considered below.

Setting the skewness coefficient $\overset{´}{\gamma }\left(p\right)={\overset{´}{\mu }}_{3}\left(p\right)/{\overset{´}{\sigma }}^{3}\left(p\right)$, 100(1−α) percent skewness‐corrected, observed information Firth penalized score CIs are found as the solutions to $\overset{´}{Z}\left(p\right)-B\overset{´}{\gamma }\left(p\right)=\pm z_{\alpha /2}$, or equivalently, as the solutions to

(11) $$\begin{array}{c} {\left[\overset{´}{Z}\left(p\right)-B\overset{´}{\gamma }\left(p\right)\right]}^{2}=\frac{{\left(\sum_{i=1}^{d}N_{i}\left[\frac{x_{i}-n_{i}\left(1-Q_{i}\right)}{n_{i}\left(1-Q_{i}\right)}\right]W_{i}\left(p\right)-B\;\;\frac{\sum_{i=1}^{d}\frac{m_{i}^{3}n_{i}Q_{i}\left(2Q_{i}-1\right)}{{\left(1-Q_{i}\right)}^{2}}W_{i}^{3}\left(p\right)}{\sum_{i=1}^{d}\frac{m_{i}^{2}n_{i}Q_{i}}{1-Q_{i}}W_{i}^{2}\left(p\right)}\right)}^{2}}{\sum_{i=1}^{d}\frac{m_{i}^{2}n_{i}Q_{i}}{1-Q_{i}}W_{i}^{2}\left(p\right)}=z_{\alpha /2}^{2}. \end{array}$$

As with Equation (10), when d=1, this reduces to the corresponding estimating equation for the expected information interval in Equation (4), as the $W_{1}\left(p\right)$ terms cancel.

Should other inference based on S(p) or i(p) be of interest, direct computation or algebraic expansion of the bracketed term in ${\overset{´}{\sigma }}^{2}\left(p\right)$ directly gives both the variance of i(p) as

(12) $$\begin{array}{c} \sigma_{i(p)}^{2}=\text{Var}\left[i\left(p\right)\right]=\frac{1}{{\left(1-p\right)}^{4}}\sum_{i=1}^{d}\frac{m_{i}^{2}n_{i}Q_{i}}{1-Q_{i}}{\left[\frac{\left(m_{i}+1\right)Q_{i}-1}{1-Q_{i}}\right]}^{2}\; \end{array}$$

and the covariance of S(p) and i(p) as

$$\begin{array}{c} \text{Cov}\left[S\left(p\right),i\left(p\right)\right]=\frac{1}{{\left(1-p\right)}^{3}}\sum_{i=1}^{d}\frac{m_{i}^{2}n_{i}Q_{i}}{1-Q_{i}}\left[\frac{\left(m_{i}+1\right)Q_{i}-1}{1-Q_{i}}\right]. \end{array}$$

We use $\sigma_{i(p)}^{2}$ below in examination of performance of expected and observed CIs. Finally, one expects that for observed values of $x_{i}$ near $E[x_{i}]$, we should have i(p)≈I(p), and so $\overset{´}{Z}\left(p\right)\approx \overset{˘}{Z}\left(p\right)$. This is readily seen to be true, noting

$$\begin{array}{ccc} i(p)-I(p) & = & \frac{1}{{\left(1-p\right)}^{3}}\sum_{i=1}^{d}N_{i}\left[\frac{x_{i}-n_{i}\left(1-Q_{i}\right)}{n_{i}\left(1-Q_{i}\right)}\right]\left[\frac{\left(m_{i}+1\right)Q_{i}-1}{1-Q_{i}}\right], \end{array}$$

which is approximately 0 when the $x_{i}\approx E\left[x_{i}\right]=n_{i}\left(1-Q_{i}\right)$, for each i=1,2,⋯,d. Unsurprisingly then, observed and expected information CIs should be similar when observations are near their expected values.

## 4 Pooled Testing Examples

Use of pooled samples is commonplace in entomological applications, in particular for virus infection prevalence estimation in mosquitoes. In these applications, infection prevalence is expected to be low, often below 50/1000, and so testing of pooled rather than of individual mosquitoes is economical. We illustrate the CIs developed for two applications of this nature.

### 4.1 Mosquito Larval Development and Yellow Fever Virus Strain Impact on Transovarial Transmission

Experimental results used to evaluate the roles of larval development duration and virus strain on the prevalence of transovarial transmission of two yellow fever virus strains isolated from either *Alouatta seniculus* (Colombian red howler monkey) or *Haemagogus* mosquitoes were reported by Walter et al. (1980). To illustrate the CIs derived, we use the subset of these data from the virus strain isolated from *A. seniculus* that were tested at larval development interval 11–15 days. Writing $N:m_{x}^{n}$ to annotate the design and results, with each pool size m together with the number x of n pools that were positive, these data were: $2421:80_{1}^{3}\;100_{3}^{12}\;103_{0}^{1}\;111_{1}^{2}\;115_{0}^{1}\;116_{0}^{1}\;123_{0}^{1}\;150_{1}^{1}\;152_{0}^{1}$. To summarize, there were 2421 individuals allocated into nine different pool sizes ranging 80–152 in 23 pools, of which six were positive.

The expected information Firth‐corrected point estimate of the prevalence of yellow fever virus infection in this sample was $\overset{˘}{p}=$ 0.0028035 or 2.8035 per 1000, and this value is the maximizer of the expected information Firth penalized log‐likelihood $\overset{˘}{l}\left(p\right)$ in Equation (7). (Prevalence estimates are often sufficiently small in entomological applications, for example, that scaling them “per 1000” is common to enhance readability.) Using the observed information, the estimate is $\overset{´}{p}=$ 2.8018 per 1000, and this value maximizes the observed information Firth penalized log‐likelihood $\overset{´}{l}\left(p\right)$ computed from Equation (9). The MLE is $\hat{p}$ = 2.8757 per 1000.

### 4.2 WNV Mosquito Surveillance, Fort Collins, Colorado

As a component of surveillance for WNV activity in the city of Fort Collins, Colorado, USA, mosquitoes are trapped and tested weekly during the transmission season (https://www.fcgov.com/westnile). Mosquito abatement decisions are made in part using estimates of WNV infection prevalence computed from the trapping and testing results. Mosquitoes are pooled for testing according to trap and species, and traps may be combined for convenient geographic evaluation.

For illustration of the methods developed, we use observations from the year 2016 collected in week 35, for traps set in the southeast zone of the city. For the WNV vector species *Culex tarsalis*, a principle vector in the area, there were 108 individual mosquitoes collected in the 14 traps during this week of surveillance. For analysis, these were pooled and tested, with results $108:1_{1}^{2}\;2_{0}^{1}\;4_{0}^{1}\;5_{1}^{1}\;6_{0}^{2}\;10_{0}^{1}\;11_{0}^{1}\;12_{0}^{4}\;14_{0}^{1}$; in summary, the 108 individuals were grouped into 14 pools of nine different sizes (ranging 1–14), of which two were positive. For these data, $\overset{˘}{p}$ = 18.090 per 1000 and $\overset{´}{p}$ = 18.000 per 1000, while $\hat{p}$ = 18.875 per 1000.

### 4.3 Results

The standard and penalized log‐likelihood functions for the two examples are graphed in Figure 1, where the associated point estimates are indicated with vertical lines. The impact of the Firth correction, which is parameter p‐dependent, is theoretically to adjust the standard log‐likelihood by altering both the location of the maximum—which in these examples is hard to discern visually—and the shape of the curve, which is more apparent. Note that, as argued above, while the central moments of the associated score random variables derived using these functions are the same for the standard likelihood and the expected information Firth penalized likelihood, the curvatures, given the data, differ, resulting in the apparent shape differences seen in the figure. For the Walter et al. (1980) data, the expected and observed information‐based penalized likelihood estimates differ little, so that the lines representing them in the graph are overlaid, while those for the Fort Collins data are also close, but their difference is just visible at this scale.

**FIGURE 1:**
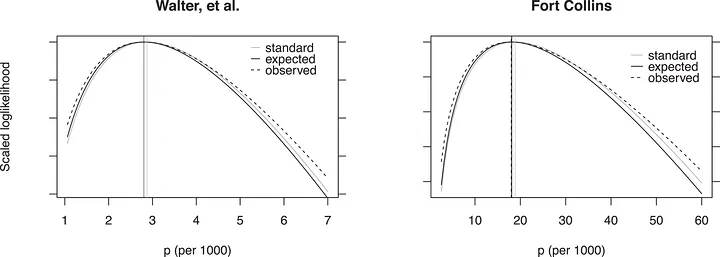
Standard and penalized log‐likelihood functions for the Walter et al. (1980) (left panel) and Fort Collins (right panel) data. Associated point estimates (log‐likelihood maximizers) are indicated with vertical lines.

In Table 1 we list the CIs developed above, introduce simplified labels used in discussions of the evaluation below, and apply them to the Walter et al. (1980) and Fort Collins data. Differences among the CIs are relatively minor in the Walter et al. (1980) example, differing negligibly for the CI limits between the expected and observed information intervals, while the intervals differ more between the score intervals and those skewness‐corrected. For the Fort Collins data, differences are again more pronounced by skewness correction, particularly for the lower limits, than whether expected or observed information is used. Below we investigate, using this example, when expected and observed information intervals may be anticipated to differ.

**TABLE 1:** Penalized likelihood‐based confidence intervals developed, with labels used in subsequent discussion and with observed values computed for the Walter et al. and Fort Collins examples. Estimates are per 1000 mosquitoes.

Information	CI method	Label	Equation	Walter et al.	Fort Collins
Expected	Penalized score	EPS	3	1.28−5.94	5.21−57.75
	Penalized skew‐corrected score	EPSS	4	1.16−5.91	3.46−56.87
Observed	Penalized score	OPS	10	1.28−5.93	5.20−57.45
	Penalized skew‐corrected score	OPSS	11	1.16−5.91	3.45−56.58

## 5 Numerical Evaluation

We compare the performances of the CIs presented by computing exact measures of coverage, median absolute deviation (MAD) from nominal 95% coverage, expected length, and directional noncoverage (see, e.g., Newcombe 1998). These were computed over a range of pooling designs (pool size‐sample size configurations) and over a range of correspondingly reasonable values for p, such that 0<p<ψ, with ψ set to the value of p for which the probability of all positive groups is 0.05, following Hepworth (2004). For each pooling design, CIs for each potential outcome were computed at 1000 equally spaced points in (0,ψ); exact coverage, expected length, and summary measures of performance were computed from these. For reference, coverage computations for each pooling design required $\prod_{i=1}^{d}\left(n_{i}+1\right)$ evaluations of each CI for each p. In principle, when choosing among CIs, we prioritize performance based on coverage, then expected length, and finally directional noncoverage.

Pooling designs used in the evaluations are detailed in Table 2. In this table we highlight in bold type three pooling designs with different “asymptotic” characteristics that we use to illustrate exact pooling results. Design $1000:100^{10}$ has a number of individuals (1000) grouped into few pools (10). Design $1000:5^{100}\;10^{40}\;25^{4}$ has the same number of individuals as the preceding design, but pooled into a much larger number of pools (144) of three different pool sizes, with many of the small pool size and few large‐sized pools, so this design represents relatively little pooling. Finally, design $2500:10^{10}\;50^{48}$ represents an application that might occur in larger collection entomological applications, with many larger and few smaller pools.

**TABLE 2:** Values of $\left\{n_{i}\right\}$ for the given $\left\{m_{i}\right\}$ of the pooling designs used in the performance evaluations, by the number of pool sizes and total number (N) of individuals. Total number of pools, average pool size, and ψ are also reported. Results of the three pooling designs in bold type are shown in detail in subsequent figures and tables.

		Pool size $m_{i}$								
Number of pool sizes	N	5	10	20	25	50	100	Number of pools	Mean pool size	ψ
1	100				4			4	25.0	0.025
	100		10					10	10.0	0.126
	500						5	5	100.0	0.008
	500					10		10	50.0	0.027
	500			25				25	20.0	0.103
	500		50					50	10.0	0.248
	500	100						100	5.0	0.506
	**1000**						**10**	**10**	**100.0**	**0.013**
	1000			50				50	20.0	0.133
	1000	200						200	5.0	0.569
	5000	1000						1000	5.0	0.687
2	75	5	5					10	7.5	0.182
	100		5			1		6	16.7	0.077
	100	10	5					15	6.7	0.250
	200		10			2		12	16.7	0.126
	200	8		8				16	12.5	0.211
	260		1			5		6	43.3	0.026
	275	5			10			15	18.3	0.154
	300		5			5		10	30.0	0.079
	375				5	5		10	37.5	0.039
	490		24			5		29	16.9	0.193
	520		2			10		12	43.3	0.046
	625				5	10		15	41.7	0.045
	700		35			7		42	16.7	0.221
	800		40			8		48	16.7	0.231
	980		48			10		58	16.9	0.245
	1000				20	10		30	33.3	0.078
	1000	100				10		110	9.1	0.506
	1125				5	20		25	45.0	0.052
	1250		5			24		29	43.1	0.084
	1250	10			48			58	21.6	0.239
	1470		72			15		87	16.9	0.274
	1820		7			35		42	43.3	0.104
	1960		96			20		116	16.9	0.294
	2080		8			40		48	43.3	0.112
	2300	10				45		55	41.8	0.237
	2450		120			25		145	16.9	0.309
	2500	20					24	44	56.8	0.326
	**2500**		**10**			**48**		**58**	**43.1**	**0.128**
	2500	10				49		59	42.4	0.237
	3750		15			72		87	43.1	0.157
	5000		10				49	59	84.7	0.126
	5000		10			98		108	46.3	0.129
	5000		20			96		116	43.1	0.179
	5000				100	50		150	33.3	0.132
	5000	500				50		550	9.1	0.641
	6250		25			120		145	43.1	0.196
3	200	5	5		5			15	13.3	0.183
	325	5	5		10			20	16.2	0.184
	400	5			5	5		15	26.7	0.151
	775	5			10	10		25	31.0	0.154
	1000		20		8	12		40	25.0	0.180
	1000		50		12	4		66	15.2	0.248
	**1000**	**100**	**40**		**4**			**144**	**6.9**	**0.507**
	1300				2	5	10	17	76.5	0.030
	3050	10			20	50		80	38.1	0.238
	5000		200		60	30		290	17.2	0.344
4	1000	10	10		10	12		42	23.8	0.261
	1000	20	40		12	4		76	13.2	0.350

### 5.1 Performance of CIs for Specific Examples

For the three designs set in bold type in Table 2 as just described and the design used by Walter et al. (1980) and in the Fort Collins real‐time collections, exact coverages for p∈(0,ψ) for the CIs are graphed in Figure 2. In the figure, results for each pooling design are displayed in one row of panels, with the first two panels containing, respectively, the expected and observed information Firth penalized score intervals, and the next two the corresponding skewness‐corrected intervals. Numerical summaries of these coverages are given in Table 3, where for each we report mean coverage (Coverage); median absolute deviation from nominal coverage (MAD); mean expected length; and mean noncoverage symmetry, defined as the difference in left and right noncoverage divided by their sum (see Biggerstaff 2008), all computed over (0,ψ).

**TABLE 3:** Performance over p∈(0,ψ) of three example pooling designs in bold type in Table 2 and the designs used in Walter et al., and Fort Collins real data examples. Performance measures include average coverage (percent), expected length (per 1000), and noncoverage symmetry (percent).

Design	Information	Method	Coverage	MAD	Exp. length	Non‐cov. Symmetry
$1000:100^{10}$	Expected	Score	95.17	1.09	101.05	22.11
	Observed	Score	95.17	1.09	101.05	22.11
	Expected	Skew‐Score	95.82	1.21	102.92	17.55
	Observed	Skew‐Score	95.82	1.21	102.92	17.55
$1000:5^{100}\;10^{40}\;25^{4}$	Expected	Score	94.98	0.15	44.11	−15.72
	Observed	Score	94.98	0.15	44.09	−15.75
	Expected	Skew‐Score	95.06	0.14	44.42	0.43
	Observed	Skew‐Score	95.06	0.14	44.39	0.41
$2500:10^{10}\;50^{48}$	Expected	Score	94.75	0.46	18.08	−34.46
	Observed	Score	94.73	0.46	18.04	−35.37
	Expected	Skew‐Score	95.12	0.45	18.77	0.74
	Observed	Skew‐Score	95.13	0.46	18.76	0.26
Walter et al.	Expected	Score	94.98	0.58	8.09	−3.71
	Observed	Score	94.98	0.57	8.08	−3.73
	Expected	Skew‐Score	95.26	0.71	8.48	4.99
	Observed	Skew‐Score	95.27	0.69	8.47	4.93
Fort Collins	Expected	Score	94.70	0.74	113.63	−24.46
	Observed	Score	94.65	0.73	112.14	−26.21
	Expected	Skew‐Score	95.44	0.75	121.91	3.80
	Observed	Skew‐Score	95.48	0.79	120.64	3.35

**FIGURE 2:**
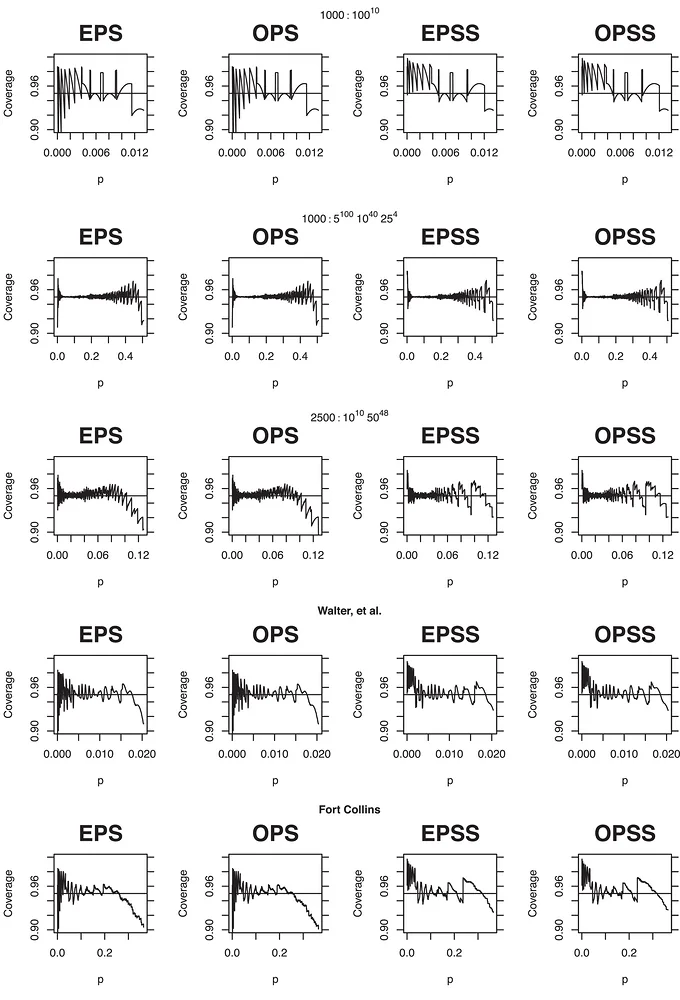
Exact coverages for the pooling designs indicated, over p∈(0,ψ). EP(S)S = expected information profile (skewness‐corrected) score; OP(S)S = observed information profile (skewnewss‐corrected) score.

Inspection of the coverage graphs in Figure 2 uncovers a few apparent tendencies with regard to the methods. As expected, the sample size impacts the variability of the coverage over p, more so the number of pools than the number of individuals. For example, for the designs in the top two rows both have 1000 individuals, but the variability in coverage for the first‐row design with 10 pools is much greater over all of p∈(0,ψ) than for the second‐row design, which has 144 pools, most of them small pools. For each of the examples, the skewness correction to the score function provides better coverage for higher values of p, seen in the graphs by noting that coverage drops with increasing p for the uncorrected score intervals, while the coverage lines stay reasonably symmetric about the nominal 95% line for the skewness corrected intervals. This symmetrization of coverage is also seen over a wider range of values for p in the large‐sample design with 2500 individuals, where for the uncorrected score intervals the coverage tends to increase with p through about (0,ψ/2), but coverage over this range is symmetric about the nominal value with skewness correction. The skewness correction also yields generally conservative coverage for relatively small values of p∈(0,ψ), compared to highly variable but roughly average nominal coverage without skewness correction.

All of these observations are consistent with previous evaluations of the impact of skewness correction to the score interval (see Hepworth 2005; Biggerstaff 2008). Perhaps the most striking conclusion when evaluating these examples is that coverage when using either expected or observed information is nearly the same. This is investigated further below.

Numerical summaries of the coverage results for these examples are shown in Table 3. The skewness‐corrected intervals have slightly higher coverage, and all above nominal 95%, while the score intervals are below nominal except for the $1000:100^{10}$ design. The skewness‐corrected intervals also are slightly more variable in their coverages, with MAD values similar or greater than the uncorrected intervals. As expected, the skewness correction does lengthen the intervals, consistent with increased coverage. Asymmetric non‐coverage of the score intervals is greatly reduced by the skewness correction, with all values of non‐coverage symmetry considerably closer to zero. In only the $1000:100^{10}$ design here does the score interval tend to “overshoot” the true value for p, as in the other examples the intervals miss low, reflected in the negative non‐coverage symmetry values.

### 5.2 Overall Performance of CIs

Shown in Figure 3 are mean coverages over p∈(0,ψ) for each design in Table 2, by CI method and information type. As with the examples of the previous section, the results regarding skewness correction are consistent with general increase in coverage, from just under nominal to just above nominal, and this agrees with previous evaluations.

**FIGURE 3:**
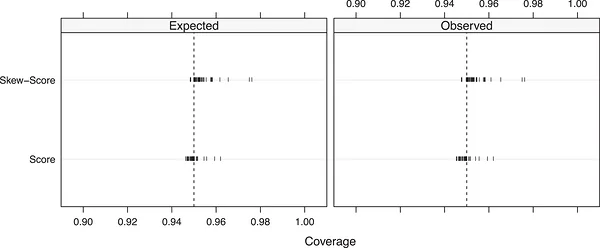
Coverages for CI method by information type for all designs in Table 2.

Differences between information type also appear to be slight, and quartiles for these distributions differ only in the fourth decimal (values not shown).

In Table 4 we report overall summary mean performance measures over all designs in Table 2. These reinforce our conclusions from the examples and the coverage graph. The skewness correction improves coverage and non‐coverage symmetry, at the understandable expense of increased interval width. Variability in coverage as measured by MAD slightly favors the observed information correction intervals, and they are also slightly shorter, on average, than their expected information counterparts. The impact of this is reflected in comparatively slight increase in coverage asymmetry.

**TABLE 4:** Summaries of CI performance, averaged over all designs in Table 2 and associated values of p∈(0,ψ). All quantities but noncoverage symmetry have been multiplied by 100 to ease readability.

Information	Method	Coverage	MAD	Exp. length	Left non‐cov.	Right non‐cov.	Non‐cov. symmetry
Expected	Score	94.99	0.6944	5.668	2.793	2.213	11.58
Observed	Score	94.98	0.6925	5.651	2.820	2.199	12.38
Expected	Skew‐Score	95.32	0.7705	5.954	2.185	2.495	−6.63
Observed	Skew‐Score	95.32	0.7709	5.922	2.183	2.496	−6.69

### 5.3 Effect of Information Type on CI Performance

As the performance evaluation has shown, the differences between the overall interval coverage properties when using either expected or observed information are slight, differing only for the examples used in our evaluation in the third or fourth decimal values for the measures used. When considering why this should be the case, we recall the comment at the end of Section 3 that the observed information penalized score statistic, $\overset{´}{Z}\left(p\right)$, should be similar to the expected information penalized score statistic, $\overset{˘}{Z}\left(p\right)$, when i(p) is near I(p). Since E[i(p)]=I(p), we therefore anticipate comparable performance of expected and observed information‐based intervals when i(p)−I(p)≈0, or the observed data values $\left\{x_{i}\right\}$ are near their expected values, $\left\{n_{i}\left(1-Q_{i}\right)\right\}$. The measures we used above to compare performance of CIs are overall, “averaging” measures which weight more likely outcomes higher, and the averaging of observed information in this sense is reflected in its overall performance and, therefore, naturally agrees with the expected information performance.

Over the range of possible data outcomes, however, we anticipate that values for the CI functions and resulting CIs will differ more as the data differ more from their expected values under the design, depending on p. Inspection of the CI functions Z(p) and $\overset{´}{Z}\left(p\right)$ and their corresponding skewness‐corrected versions indicate why this should be. The differences in the CI functions in Equations (3) and (10) and their corresponding skewness‐corrected analogs in Equations (4) and (11) are the weights $\left\{W_{i}\left(p\right)\right\}$, which derive from the development using the observed information i(p) (Equation (2)). The CI functions incorporate the data $\left\{x_{i}\right\}$ through the relative difference between the observed number of positive pools, $\left\{x_{i}\right\}$, and their expectations, $\left\{n_{i}\left(1-Q_{i}\right)\right\}$, via $\left\{\delta_{i}\equiv \left[x_{i}-n_{i}\left(1-Q_{i}\right)\right]/\left[n_{i}\left(1-Q_{i}\right)\right]\right\}$, as these CI functions derive from their interpretations and use as test statistics, and the CIs being obtained from them by the method of “test inversion.”

In the expected and observed information CI functions Z(p) and $\overset{´}{Z}\left(p\right)$, the “full weight” given $\delta_{i}$ are, respectively,

$$\begin{array}{c} \frac{N_{i}}{\sqrt{\sum_{i=1}^{d}\frac{m_{i}^{2}n_{i}Q_{i}}{1-Q_{i}}}}\;\text{and}\;\frac{N_{i}\;W_{i}\left(p\right)}{\sqrt{\sum_{i=1}^{d}\frac{m_{i}^{2}n_{i}Q_{i}}{1-Q_{i}}W_{i}^{2}\left(p\right)}}. \end{array}$$

Use the notation (·) to denote a generic, unspecified index to facilitate this discussion. The way the observed information‐derived weights $W_{\left(\cdot \right)}\left(p\right)$ are incorporated is clear from these expressions, though their impact is complex, depending nonlinearly on p and the pools sizes, $m_{\left(\cdot \right)}$. When comparing the weight assigned the data, $\delta_{\left(\cdot \right)}$, for two different pool sizes, with different indices i and j, say, their ratio for the expected information case is $N_{i}/N_{j}$, and for the observed information case it is $\left[N_{i}W_{i}\left(p\right)\right]/\left[N_{j}W_{j}\left(p\right)\right]$, the difference being the weight functions $W_{\left(\cdot \right)}\left(p\right)$. So for the observed information CIs, relative deviations $\delta_{\left(\cdot \right)}$ of observations from their respective expectation are deferentially weighted by the $W_{\left(\cdot \right)}$, which are functions of both the pool size and of complex functions of p through b(p) and the $Q_{\left(\cdot \right)}$. The corresponding weights for the $\delta_{\left(\cdot \right)}$ for the expected information do not depend on p nor on local (observed) information. Note that the $W_{\left(\cdot \right)}$ are increasing functions of $m_{\left(\cdot \right)}$ for fixed p, so that the differences $\delta_{\left(\cdot \right)}$ are weighted more heavily for larger pools at a fixed value of p. The observed information CI therefore incorporates directly weights which modify the expected information CI function by incorporating components of the observed information function.

As discussed after Equations (3) and (10), when d=1, so that there is only a single pool size, the observed information CI function $\overset{´}{Z}\left(p\right)$ reduces to the expected information CI function Z(p), as the weights $W_{1}\left(p\right)$ cancel, which is also the case with the skewness‐corrected versions of the functions. Intuitively, this agrees with the fact this case is a standard binomial, with size parameter $n_{1}$ and probability of success $1-{\left(1-Q_{1}\right)}^{m_{1}}$. The notion of an atypical, or “unexpected,” outcome for the multiple pool size case arises because a basic assumption underpinning the model is that individuals are allocated to pools independently, and so it follows that larger pools are more likely to be positive than smaller pools. Outcomes for which this is not the case are possible, if less probable, however, and it is in these cases that incorporation of observed information into the CI function via $W_{i}\left(p\right)$ may theoretically be expected to improve interval performance over the use of expected information. We illustrate and investigate this in greater detail now.

As an example, we consider differences between expected and observed information CI functions for different outcomes for the design $300:5^{10}\;50^{5}$. In the top row of Figure 4, we graph the the expected and observed skewness‐corrected penalized score estimating functions from Equations (4) and (11), respectively. The first panel in the top row shows these functions for the outcome (3,5), the second panel for the outcome (5,3), and the third panel for the outcome (8,0). The two roots of these curves give the respective CI endpoints for the given outcomes. The outcomes in this order represent a progression of increasingly less‐probable outcomes for any value of p, and in this sense (8,0) is less “expected” or “likely” than is (5,3), which is less likely than (3,5), mirrored in the corresponding expected values for $\left(x_{1},x_{2}\right)$ for any p. Correspondingly, the CI endpoints differ by an increasing amount as the outcomes become less likely.

**FIGURE 4:**
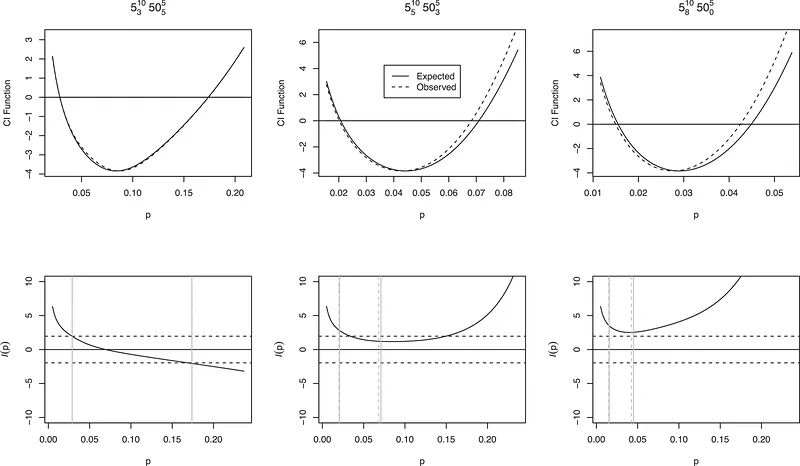
Top row: confidence interval functions for expected (solid) and observed (dashed) penalized skewness‐corrected score intervals for the $5^{10}\;50^{5}$ design for the different outcomes indicated, each with eight positive pools. Bottom row: standardized observed information functions I(p) (solid black curves) corresponding to the column‐specific design and outcome over p∈(0,ψ), with reference standard normal 95% quantiles $\pm z_{0.025}$ (dashed lines) and EPSS [OPSS] CIs (vertical solid [dashed] gray lines) for the column‐specific design and outcome.

These observations suggest that when performing an analysis, which information type to use in computation of CIs may depend on how far from “expected” or “likely” the observations are. The graphical analysis just presented could be used to evaluate differences in the CIs, but we now discuss a more objective tool to aid evaluation and inform recommendations.

As the CI functions differ by information type when i(p)−I(p)≠0, characterization of this difference as impactful for a given set of data should be useful in deciding when the observed information CI may be preferred. Above we derived an expression for $\sigma_{i(p)}^{2}$ in Equation (12), so we can compute a standardized information statistic

$$\begin{array}{c} I\left(p\right)=\frac{i(p)-I(p)}{\sqrt{\sigma_{i(p)}^{2}}}=\frac{\sum_{i=1}^{d}N_{i}\left[\frac{x_{i}-n_{i}\left(1-Q_{i}\right)}{n_{i}\left(1-Q_{i}\right)}\right]\left[\frac{\left(m_{i}+1\right)Q_{i}-1}{1-Q_{i}}\right]}{\sqrt{\sum_{i=1}^{d}\frac{m_{i}^{2}n_{i}Q_{i}}{1-Q_{i}}{\left[\frac{\left(m_{i}+1\right)Q_{i}-1}{1-Q_{i}}\right]}^{2}}} \end{array}$$

and I(p)∼N(0,1), asymptotically, for each p. As with the CI functions above, when d=1, this expression reduces by cancellation of the term in square brackets. The result in this case is that I(p)=Z(p), a reflection of the fact that this case is simply a standard binomial, for which there are cannot be any “unexpected” results that in cases that d>1 result from outcomes relatively improbable under the pooling design, for a given p.

With given data for the case d>1, then, one may compute I(p) and observe for which, if any, values of p the observed information is significantly different from expected, reflecting the underlying data's positive pool configuration under the design. Since I(p) is a function of p, which is unknown, evaluation may be guided by consideration of the whole range (0,ψ) for the given design.

An illustration of this is shown in the bottom row of Figure 4. Each panel in this row shows I(p) plotted against p∈(0,ψ), and horizontal, dashed lines are included at the standard normal quantiles $\pm z_{0.025}$ to indicate the significance threshold for comparison of i(p) and I(p). Vertical gray lines indicate the expected (solid) and observed (dashed) information CIs. The first panel corresponds to reasonably expected outcomes for the given design ($5^{10}\;50^{5}$) over a large range of p, with I(p) within the significance bounds across roughly p∈(0.03,0.17), which also corresponds closely to both CI limits for both information types in this example. In the middle panel, for which we saw above that the CI endpoints differed measurably by information type, we see that I(p) is still within the significance bounds for much of p∈(0.035,0.15), though near the left endpoint of the intervals there are some values for p∈(0.02,0.035) for which I(p) is outside the bounds. In the most extreme example shown in the third panel, for no values of p∈(0,ψ) is I(p) within the bounds $\pm z_{0.025}$, indicating that for this “unlikely” result (8 out of 10 positive small pools, and 0 out of 5 positive large pools) the observed information differs significantly from expected. This is reflected in the markedly differing CIs in the panel in the top right of Figure 4.

Application of these ideas to the Fort Collins data example is shown in Figure 5. We see in this real‐world data example, even though the 3 positive pools are all in the smaller pools, which is to be less expected generally, the observed information does not appear to differ from expected except in a small interval just inside the lower CI limits. This is reflected in the fact that the CI curves (left panel) nearly coincide, and so the resulting CIs are nearly the same (Table 1).

**FIGURE 5:**
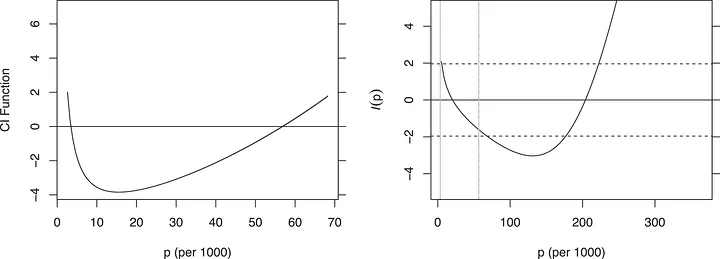
Fort Collins example CI function analyses. Left panel: expected (solid) and observed (dashed) information CI functions. Right panel: I(p) plotted against p∈(0,ψ=0.365/1000), with reference significance values $\pm z_{0.025}$ (dashed lines) and expected (vertical gray) and observed (vertical dashed) CIs. Note that because the CI functions are nearly coincident, only the expected information curve and line are clearly visible, as they overlie the observed information curve and line.

Identification of when the CIs are likely to differ by information type may be interesting and useful in a particular application, but performance of the intervals in the case of such difference is not characterized in the overall results summarized above. To decide whether the expected or observed information‐based intervals exhibit better performance in the cases of “likely” and “unlikely” results, as indicated by a significant value for I(p) under a fixed design, we undertook an evaluation of coverage properties by evaluating performance for these cases separately. For a fixed design, let X be the set of all possible outcomes and define the “likely” and “unlikely” outcome sets at p by the partition of X into sets $L_{\alpha }\left(p\right)=\left\{x\in X:|I\left(p\right)|\leq z_{1-\alpha /2}\right\}$ and $U_{\alpha }\left(p\right)=\left\{x\in X:|I\left(p\right)|>z_{1-\alpha /2}\right\}$, respectively, with 0<α<1. To evaluate potential differences in performance by information type not captured in the overall analyses presented above, we evaluate coverage performance at α=0.05 of the observed and expected information skewness‐corrected score intervals as above with the overall evaluations over each of these sets separately.

For example, for the illustrative pooling design $300:5^{10}\;50^{5}$ used above, over p∈(0.237/1000,ψ=0.237), the mean coverage over $U_{0.05}\left(p\right)$ using expected information (EPSS) is 19.55% and using observed information (OPSS) is 20.58%. Thus, in this example, using observed information provides an improvement in coverage for the unlikely data outcomes. Similarly, restricting to $L_{0.05}\left(p\right)$, coverage is 98.98% for EPSS and 98.88% for OPSS, again improved—closer to nominal—coverage when using observed information. For reference, the overall coverages for this example are 95.05% for EPSS and 95.00% for OPSS.

Rather than report a full evaluation over $L_{0.05}\left(p\right)$ and $U_{0.05}\left(p\right)$ over all pooling designs in Table 2 as in the overall analysis, we focus on coverage alone, as the results sufficiently inform subsequent recommendations: Average coverage over all pooling designs for $L_{0.05}\left(p\right)$ sets was 98.69% when using expected information and was 98.68% when using observed information, and average coverage over all designs for $U_{0.05}\left(p\right)$ sets when using expected information was 34.24% and was 34.67% when using observed information. Of note, over the 59 pooling designs evaluated, for $U_{0.05}\left(p\right)$, all coverages using observed information were equal to (11/59, 18.6%) or closer to (48/59, 81.4%) nominal than when expected information was used. And in this case, the average relative increase in coverage was 1.16%. Comparatively, for $L_{0.05}\left(p\right)$, 11/59 (18.6%) had equal coverage, while 41/59 (69.5%) were closer to nominal when observed information was used, and 7/59 (11.9%) were closer to nominal when expected information was used.

### 5.4 Recommendations

Consistent with previous evaluations, without skewness correction, coverage of the score intervals is below nominal and non‐coverage is markedly asymmetric. The skewness‐corrected intervals exhibit mean coverage at least nominal but not generally too conservative and have non‐coverage nearly symmetric, and so they are recommended. When there is just one pool size, since the observed information (score‐based) CIs reduce to the expected information CIs, consistent with previous literature (e.g., Hepworth 2005) the recommendation is to use the (expected information) skewness‐corrected score interval, with the simple expression Equation (5) providing a convenient form for computation.

Over the designs in our evaluation, as expected there was little difference in overall performance between the expected and observed information‐based intervals, based on the averaging criteria used. When investigating specific cases for which the skewness‐corrected intervals differed by information type, we showed how the standardized information statistic I(p) can be used to identify when i(p)≠I(p) significantly, and so the CIs are expected to differ. In our detailed evaluation of the coverage performance of both expected and observed information skew‐corrected score intervals, we found that coverage was at least as close to nominal when using observed information for unlikely data outcomes, and over 80% of the time these were closer to nominal, resulting in higher average coverage for these situations. For likely data outcomes, when observed information and expected information do not differ significantly, coverage performance was nearly the same between the information types, but even here using observed information resulted in more designs (70%) for which coverage was closer to nominal.

Given the overall performances of the expected and observed information intervals were nearly the same, and the coverage performance of observed information intervals was better for unlikely data outcomes (over $U_{0.05}\left(p\right)$) and nearly the same or closer to nominal for likely data outcomes (over $L_{0.05}\left(p\right)$), we recommend the observed information, skewness‐corrected penalized likelihood intervals (OPSS) for general use.

As a result of this recommendation, in formal presentation we would report 95% CI 1.16–5.91 per 1000 for the Walter et al. example and 95% CI 3.45–56.58 per 1000 for the Fort Collins example.

## 6 Discussion

Taking as a starting point the documented improved performance in point estimation provided by Firth's corrected score, we sought to develop CIs in a unified inferential framework by interpreting the Firth correction estimation function in a penalized likelihood context. This approach has been adopted in logistic regression analyses (e.g., Heinze and Schemper 2002), following Firth's development for generalized linear models (GLMs) with the canonical link function and illustration for logistic regression (Firth 1993). Except for the single pool size case, which is the standard binomial model, our model does not fit into the GLM framework with a canonical link function, so in the framework of our model we developed the corresponding penalized likelihoods and computed associated score‐based CIs using the familiar likelihood‐based motivations. We restricted focus to score methods since previous evaluations have consistently shown them to be preferred. We presented readily interpretable formulas for the CI functions, being based on score test inversions, and we gave simple expressions for the CIs when there was a single pool size, giving an extension of the result in Laud (2017). Finally, we evaluated the performance of these intervals over a range of pooling designs and for particular examples from the entomological and public health literature and practice.

Deriving the expected information penalized score‐based CIs, we showed the key result that these intervals are computationally equivalent to the standard likelihood score intervals, including when applying the skewness‐correction of the score statistic as introduced by Bartlett (1953) and further developed by Gart (1991). Review of the derivations for this case shows that this result follows because the Firth correction to the score, −I(p)b(p), is not data‐dependent, so that central moments agree with the standard likelihood. Firth (1993) showed that for non‐exponential family models, as here, one may expect improved, second‐order performance in point estimation using instead the observed information in the score correction, and while this has not been formally evaluated for this model, computation is straightforward using numerical root‐finding routines, as illustrated here. Use of this estimator is reasonably justified by this theoretical result, previous work demonstrating the superior performance of the Firth estimator using expected information, and its coordinated use with the observed information‐based CI recommended here to be within a consistent inferential framework. In adapting this information type for CIs, we derived the mean, variance, and skewness of the corrected score function since the central moments do not agree with those of the standard score statistic. We then used these moments to standardize the corrected score function for use in penalized likelihood, score‐based CIs.

We derived the variance of the observed information, used it to compute the standardized information statistic I(p), and used this to characterize when expected and observed information CIs would differ. This led to a more detailed evaluation of coverage performance by information type by partitioning the outcome set for a fixed design into sets of likely and unlikely outcomes. Results of this evaluation showed improved performance when using the observed information type for both likeliness classes, leading to its general recommendation.

We have assumed throughout the presentation that the positivity status of pools was determined using a perfect evaluation, test, or assay. Extension of the results to imperfect test by including explicitly sensitivity and specificity parameters should be direct, following, for example, the work on the use of Firth's correction for point estimation in Hepworth and Biggerstaff (2021) in this context.

Comparison of the CIs here with competitors derived using different approaches, such as other likelihood CIs in the frequentist paradigm or Bayesian intervals, where for standard binomial inference the Jeffreys prior has shown good frequentist properties, is beyond the scope of this work. (Researchers who prefer not to mix frequentist and Bayesian methods may cast the Bayesian approach in a frequentist penalized likelihood framework for comparison to our developments here, perhaps using, then, a different prior than that induced by the Firth correction, such as the Jeffreys prior.) Given the general performance of both the expected and observed information CIs developed here, we expect that qualitative comparison with other methods would likely result in conclusions similar to those already available in existing literature comparing the score and skewness‐corrected CIs to others (see, e.g., Hepworth 2005).

When anticipating use of the CIs for application, researchers may first wish to consider a variety of pooling designs, depending on the application and resources available for sample collection and testing. In such evaluation, we encourage researchers to evaluate the pooling designs being considered over the range of anticipated prevalence, in an effort to choose a design that should yield as precise an inference as possible. In particular, one must try to ensure at least some negative pools, and a certain balance in positive and negative pools is desirable, else these methods of likelihood‐based pooled inference become noninformative. (See, for example, Schaarschmidt (2007).)

Current recommendations for inference when estimating a proportion from pooled samples are to use standard likelihood inference that requires a correction for acceptable point estimation, with interval estimation following from score methods. The penalized likelihood framework developed here unifies these established and recommended methods of point and interval estimation into a consistent inferential approach, in which use of observed information is recommended.

## Funding

The authors have nothing to report.

## Disclosure

The content and conclusions are those of the authors and do not necessarily represent the official position of the Centers for Disease Control and Prevention (CDC). Permission to use data from the City of Fort Collins, Natural Areas Department, was granted in writing by Mr. Matt Parker, Ecological Stewardship Manager. All data used are reported in the paper.

## Conflicts of Interest

The authors declare no conflicts of interest.

## Open Research Badges

This article has earned an Open Data badge for making publicly available the digitally‐shareable data necessary to reproduce the reported results. The data is available in the Supporting Information section.

This article has earned an open data badge “**Reproducible Research**” for making publicly available the code necessary to reproduce the reported results. The results reported in this article could fully be reproduced.

## Supporting information


**Supporting File:** bimj70185‐sup‐0001‐Data.zip.

## Data Availability

The data that support the findings of this study are available in the Supporting Information of this article.
